# ﻿ *Stromatolinea*, a new diatrypaceous fungal genus (Ascomycota, Sordariomycetes, Xylariales, Diatrypaceae) from China

**DOI:** 10.3897/mycokeys.108.126712

**Published:** 2024-09-04

**Authors:** Kamran Habib, Xin Zhou, Wenyu Zeng, Xu Zhang, Hongmin Hu, Qianzhen Wu, Lili Liu, Yan Lin, Xiangchun Shen, Jichuan Kang, Qirui Li

**Affiliations:** 1 State Key Laboratory of Functions and Applications of Medicinal Plants, Guizhou Medical University, Guiyang, 550025, China; 2 The High Efficacy Application of Natural Medicinal Resources Engineering Centre of Guizhou Province (The Key Laboratory of Optimal Utilization of Natural Medicine Resources), School of Pharmaceutical Sciences, Guizhou Medical University, Gui’an New District, 561113, China; 3 Department of Botany, Khushal Khan Khattak University, Karak, KP, Pakistan; 4 Immune Cells and Antibody Engineering Research Centre of Guizhou Province/Key Laboratory of Biology and Medical Engineering, Guizhou Medical University, Gui’an New District, 561113, China; 5 Engineering and Research Centre for Southwest Bio-Pharmaceutical, Resources of National Education Ministry of China, Guizhou University, Guiyang, China

**Keywords:** 1 new genus, 4 new species, bambusicolous fungi, fungal systematics, Guizhou Province

## Abstract

Molecular phylogeny and morphological characteristics of collections of diatrypaceous fungi from Guizhou Province, China, lead to the establishment of a new genus, *Stromatolinea*, and the identification of four new species and two new combinations. The taxa were found growing on the dead culms of *Phyllostachys* bamboo. The new genus is distinguished by its well-developed, discrete linear stromata with yellow interior tissue and allantoid subhyaline ascospores. The newly described species are *Stromatolineagrisea*, *S.guizhouensis*, *S.hydei*, and *S.xishuiensis*. Additionally, two new combinations, *Stromatolinealinearis* and *S.phaselina*, are proposed based on comparative analysis and morphology. Phylogenetic analyses were conducted using ITS and TUB2 sequences. The study includes comprehensive morphological descriptions, illustrations, and a phylogenetic tree depicting the placement of the new taxa.

## ﻿Introduction

In recent years, several new genera within Diatrypaceae have been reported through a combination of morphological characteristics and multi-locus phylogeny. Currently, the family is represented by 27 genera, i.e., *Allocryptovalsa*, *Allodiatrype*, *Alloeutypa*, *Anthostoma*, *Cryptosphaeria*, *Cryptovalsa*, *Diatrypasimilis*, *Diatrype*, *Diatrypella*, *Dothideovalsa*, *Echinomyces*, *Endoxylina*, *Eutypa*, *Eutypella*, *Halocryptosphaeria*, *Halocryptovalsa*, *Halodiatrype*, *Leptoperidia*, *Libertella*, *Monosporascus*, *Neoeutypella*, *Paraeutypella*, *Pedumispora*, *Peroneutypa*, *Pseudodiatrype*, *Quaternaria*, and *Rostronitschkia* (Hyde et al. 2020; Konta et al. 2020; Dissanayake et al. 2021; Long et al. 2021; Samarakoon et al. 2022; Wijayawardene et al. 2022; Chen et al. 2023; Ma et al. 2023).

Diatrypaceae species are distributed worldwide and are commonly found on deadwood and the bark of various plant species. The family is characterized by black or dark brown, immersed to erumpent, pseudostromatic or eustromatic stromata, polysporous or 8-spored asci, hyaline to light brown allantoid ascospores, and a libertella-like asexual morph (Senanayake et al. 2015; Wijayawardene et al. 2017).

Bamboo, as the largest member of the grass family Poaceae, plays an important role in local economies worldwide, being distributed across diverse climates, from cold mountainous regions to hot tropical areas. China boasts plentiful bamboo resources, with its bamboo species constituting over 50% of the world’s total (Liu et al. 2018). There are more than 1300 fungal species associated with bamboo, including 150 basidiomycetes, 800 ascomycetes. The taxonomic placements of bamboo-associated ascomycetous fungi are highly diverse, comprising over 1,150 species, in 120 families and 400 genera (Dai et al. 2018; Hyde et al. 2023). Jiang et al. (2022) reported 512 bambusicolous ascomycetous taxa from China, associated with 16 bamboo genera, representing more than one-third of the known bambusicolous ascomycetes in the world.

In an investigation into the diversity of bambusicolous fungi in Guizhou, China, four previously undescribed species of diatrypaceous fungi were discovered. Morphological analyses revealed their close affinity to the genus *Alloeutypa*. However, phylogenetic analyses did not support their placement within this genus. Following detailed morphological examinations and comparative analyses, we propose a new genus, *Stromatolinea*, within the family Diatrypaceae, which includes four new species and one new combination. The findings contribute significantly to the understanding of diatrypaceous fungal diversity and taxonomy.

## ﻿Materials and methods

### ﻿Sample collection and morphological study

The specimens of bamboo were collected during surveys conducted in the Guizhou province, China. All related habitat information, including details about elevation, climatic conditions, and geographical features, was recorded. The photos of the collected materials were taken using a Canon G15 camera (Canon Corporation, Tokyo, Japan). Materials were placed in paper bags and were taken to the lab for examination. To preserve the freshness of the specimens, they were dried at room temperature. The dried specimens were carefully labeled and stored in an ultra-low freezer at –80 °C for one week to eliminate any insects and their eggs. After this preparation, the specimens were ready for both morphological and molecular studies.

Macroscopic characteristics were observed under an Olympus SZ61 stereomicroscope and photographed with a Canon 700D digital camera fitted to a light microscope (Nikon Ni). Morphological characteristics of specimens were examined, and photomicrographs were taken as described in Senanayake et al. (2020). Materials were mounted in water for anatomical examination and added Melzer’s reagent where necessary. More than 30 ascospores and 30 asci were measured using the Tarosoft image framework (v. 0.9.0.7). Images were arranged using Adobe Photoshop CS6 (Adobe Systems, USA).

Isolates were derived by single spore isolation following the method of Chomnunti et al. (2014). Germinating spores were observed with a Stereo Zoom microscope and transferred to potato dextrose agar (PDA; 39 g/l distilled water, Difco potato dextrose). The cultures were incubated at 25–30 °C for 1–4 weeks, with frequent observations. Cultural characteristics, such as mycelium colour, shape, texture and growth rate, were recorded after incubating at 25 °C under normal light for a week.

Herbarium materials were deposited at the herbarium of Guizhou Medical University (GMB) and the Herbarium of Cryptogams, Herbarium of Kunming Institute of Botany, Chinese Academy of Sciences (**KUN-HKAS**), and living cultures were deposited at the Guizhou Medical University Culture Collection (**GMBC**).

### ﻿DNA extraction, PCR amplification, and sequencing

Fungal DNA was directly extracted from the contents of stromata and perithecia using the BIOMIGA fungus genomic DNA extraction kit, following the manufacturer’s instructions. The DNA samples were stored at –20 °C. Internal transcribed spacers (ITS), and β-tubulin (TUB2), were amplified by PCR with primers ITS1/ITS4 (White et al. 1990; Gardes and Bruns 1993), and T1/T22 (Glass and Donaldson 1995; O’Donnell and Cigelnik 1997), respectively. The components of a 25 μL volume PCR mixture was: 9.5 μL of double distilled water, 12.5 μL of PCR Master Mix, 1 μL of each primer and 1 μL of template DNA. Qualified PCR products were checked through 1.5% agarose gel electrophoresis stained with GoldenView, and sent to Sangon Co., China, for sequencing (Xie et al. 2020).

### ﻿Phylogenetic analyses

All newly generated sequences from this study were deposited in GenBank (https://www.ncbi.nlm.nih.gov/; accessed on March 28, 2024; Table 1). These sequences were compared with each other and all the known sequences in the GenBank by using the BLAST algorithm for identification. The sequences retrieved from open databases originated from Li et al. (2023), Ma et al. (2023), and the BLASTn results of close matches and other Diatrypaceae representatives. The molecular phylogeny was inferred from a combined dataset of ITS and TUB2 sequences. Sequences were aligned using the MAFFT v.7.110 online programme (Katoh et al. 2019) with the default settings, respectively. Alignment was adjusted manually using BioEdit v.7.0.5.3 (Hall 1999) where necessary. The start and end of alignment were trimmed to nearly equal number of sites for all sequences. The combined sequence data was used to perform maximum likelihood (ML) and Bayesian inference analysis (BI). The ML analysis was implemented in RAxML v.8.2.12 using the GTRGAMMA substitution model with 1,000 bootstrap replicates (Stamatakis 2014).

**Table 1.** GenBank Accession Numbers used in this study. The newly generated sequences are marked bold. T indicates type strain.

Species	Isolate/specimen voucher	Reference	ITS	β-tubulin
*Allocryptovalsacastaneae*	CFCC52428^T^	Zhu et al. 2021	MW632945	MW656393
*Allocryptovalsacastaneicola*	CFCC52432^T^	Zhu et al. 2021	MW632947	MW656395
*Allocryptovalsacryptovalsoidea*	HVFIG02	Trouillas et al. 2011	HQ692573	HQ692524
*Allocryptovalsaelaeidis*	MFLUCC 15-0707^T^	Konta et al. 2020	MN308410	MN340296
*Allocryptovalsaelevata*	WA08CB	Trouillas et al. 2011	HQ692619	HQ692523
*Allocryptovalsapolyspora*	MFLUCC 17–0364^T^	Senwanna et al. 2017	MF959500	MG334556
*Allocryptovalsarabenhorstii*	GMB0416	Li et al. 2023	OP935171	OP938733
*Allocryptovalsasichuanensis*	HKAS 107017^T^	Samarakoon et al. 2022	MW240633	MW775592
*Allocryptovalsaxishuangbanica*	KUMCC 21-0830	Maharachchikumbura et al. 2022	ON041128	ON081498
*Allocryptovalsaxishuangbanica*	GMB0417	Li et al. 2023	OP935176	OP938739
*Allodiatrypealbelloscutata*	IFRD 9100 ^T^	Li et al. 2022	OK257020	NA
*Allodiatrypearengae*	MFLUCC 15-0713^T^	Konta et al. 2020	MN308411	MN340297
*Allodiatrypeelaeidicola*	MFLUCC 15-0737a	Konta et al. 2020	MN308415	MN340299
*Allodiatrypeelaeidis*	MFLUCC 15-0708a	Konta et al. 2020	MN308412	MN340298
*Allodiatrypetaiyangheensis*	IFRDCC2800^T^	Li et al. 2022	OK257021	OK345036
*Allodiatrypethailandica*	MFLUCC 15-3662^T^	Li et al. 2016	NR164240	NA
*Allodiatrypetrigemina*	FCATAS 842	Peng et al. 2021	MW031919	MW371289
*Alloeutypaflavovirens*	E48C	Rolshausen et al. 2006	AJ302457	DQ006959
*Alloeutypamilinensis*	FCATAS4309^T^	Ma et al. 2023	OP538689	OP557595
*Alloeutypamilinensis*	FCATAS4382	Ma et al. 2023	OP538690	OP557596
*Anthostomadecipiens*	IPV-FW349	Unpublished	AM399021	AM920693
*Anthostomadecipiens*	JL567	Luque et al. 2012	JN975370	JN975407
Cryptosphaeriaeunomiavar.eunomia	C1C	Acero et al. 2004	AJ302417	NA
Cryptosphaeriaeunomiavar.fraxini	C5C	Acero et al. 2004	AJ302421	NA
*Cryptosphaerialigniota*	CBS 273.87	Acero et al. 2004	KT425233	KT425168
*Cryptosphaeriapullmanensis*	ATCC 52655	Trouillas et al. 2015	KT425235	KT425170
*Cryptosphaeriasubcutanea*	DSUB100A	Trouillas et al. 2015	KT425189	KT425124
*Cryptosphaeriasubcutanea*	CBS 240.87^T^	Trouillas et al. 2015	KT425232	KT425167
*Cryptovalsaampelina*	A001	Trouillas et al. 2010	GQ293901	GQ293972
*Cryptovalsaampelina*	DRO101	Trouillas et al. 2010	GQ293902	GQ293982
*Cryptovalsaelevata*	CBS 125574	Vu et al. 2019	MH863711	NA
*Diatrypebetulaceicola*	FCATAS 2725^T^	Yang et al. 2022	OM040386	OM240966
*Diatrypebetulae*	CFCC52416^T^	Zhu et al. 2021	MW632943	MW656391
*Diatrypebetulae*	GMB0426	Li et al. 2023	OP935181	OP938750
*Diatrypebullata*	UCDDCh400	Rolshausen et al. 2006	DQ006946	DQ007002
*Diatrypecamelliae-japonicae*	GMB0427^T^	Li et al. 2023	OP935172	OP938734
*Diatrypecamelliae-japonicae*	GMB0428	Li et al. 2023	OP935173	OP938735
*Diatrypecastaneicola*	CFCC52425^T^	Zhu et al. 2021	MW632941	MW656389
*Diatrypedisciformis*	GNA14	Senanayake et al. 2015	KR605644	KY352434
*Diatrypedisciformis*	D21C	Acero et al. 2004	AJ302437	NA
*Diatrypeenteroxantha*	HUEFS155114	de Almeida et al. 2016	KM396617	KT003700
*Diatrypeenteroxantha*	HUEFS155116	de Almeida et al. 2016	KM396618	KT022236
*Diatrypeenteroxantha*	GMB0433	Li et al. 2023	OP935170	OP938736
*Diatrypelancangensis*	GMB0045^T^	Long et al. 2021	MW797113	MW814885
*Diatrypelancangensis*	GMB0046	Long et al. 2021	MW797114	MW814886
*Diatrypelarissae*	FCATAS 2723^T^	Yang et al. 2022	OM040384	OM240964
*Diatrypelijiangensis*	MFLU 19-0717^T^	Thiyagaraja et al. 2019	MK852582	MK852583
*Diatrypepalmicola*	MFLU 15-0040^T^	Liu et al. 2015	NR185365	NA
*Diatrypepalmicola*	MFLU 15-0041	Liu et al. 2015	KP744439	NA
*Diatrypequercicola*	CFCC52418^T^	Zhu et al. 2021	MW632938	MW656386
*Diatryperubi*	GMB0429^T^	Li et al. 2023	OP935182	OP938740
*Diatryperubi*	GMB0430	Li et al. 2023	OP935183	OP938741
*Diatrypespilomea*	D17C	Acero et al. 2004	AJ302433	NA
*Diatrypestigma*	DCASH200	Trouillas et al. 2010	GQ293947	GQ294003
*Diatrypeundulata*	D20C	Acero et al. 2004	AJ302436	NA
*Diatrypellaatlantica*	HUEFS 136873	de Almeida et al. 2016	KM396614	KR259647
*Diatrypellaatlantica*	LCM 888.01	Unpublished	MF495421	
*Diatrypellabanksiae*	CPC 29118 ^T^	Crous et al. 2016	KY173402	NA
*Diatrypellabetulae*	CFCC52406^T^	Zhu et al. 2021	MW632931	MW656379
*Diatrypellabetulicola*	CFCC52411^T^	Zhu et al. 2021	MW632935	MW656383
*Diatrypelladelonicis*	MFLUCC 15-1014^T^	Hyde et al. 2019	MH812994	MH847790
*Diatrypelladelonicis*	MFLU 16-1032	Hyde et al. 2019	MH812995	MH847791
*Diatrypellaelaeidis*	MFLUCC 15-0279^T^	Konta et al. 2020	MN308417	MN340300
*Diatrypellafatsiae-japonica*	GMB0422^T^	Li et al. 2023	OP935184	OP938744
*Diatrypellafatsiae-japonica*	GMB0423	Li et al. 2023	OP935185	OP938745
*Diatrypellafavacea*	380	de Almeida et al. 2016	KU320616	NA
*Diatrypellafavacea*	DL26C	Unpublished	AJ302440	NA
*Diatrypellafrostii*	UFMGCB 1917	Vieira et al. 2011	HQ377280	NA
*Diatrypellaguiyangensis*	GMB0414^T^	Li et al. 2023	OP935188	OP938742
*Diatrypellaguiyangensis*	GMB0415	Li et al. 2023	OP935189	OP938743
*Diatrypellaheveae*	MFLUCC 15-0274	Konta et al. 2020	MN308418	MN340301
*Diatrypellaheveae*	MFLUCC 17-0368^T^	Senwanna et al. 2017	MF959501	MG334557
*Diatrypellahubeiensis*	CFCC 52413^T^	Zhu et al. 2021	MW632937	MW656385
*Diatrypellairanensis*	KDQ18^T^	Mehrabi et al. 2015	KM245033	KY352429
*Diatrypellalongiasca*	KUMCC 20-0021^T^	Dissanayake et al. 2021	MW036141	MW239658
*Diatrypellamacrospora*	KDQ15	Mehrabi et al. 2016	KR605648	KY352430
*Diatrypellaoregonensis* (*Diatrypeoregonensis*)	DPL200	Trouillas et al. 2010	GQ293940	GQ293999
*Diatrypellaoregonensis* (*Diatrypeoregonensis*)	CA117	Trouillas et al. 2010	GQ293934	GQ293996
*Diatrypellapseudooregonensis*	GMB0039	Long et al. 2021	MW797115	MW814888
*Diatrypellapseudooregonensis*	GMB0041^T^	Long et al. 2021	NR174917	MW814890
*Diatrypellapulvinata*	H048	de Almeida et al. 2016	FR715523	FR715495
*Diatrypellapulvinata*	DL29C	Unpublished	AJ302443	NA
*Diatrypellatectonae*	MFLUCC 12-0172a^T^	Shang et al. 2017	KY283084	NA
*Diatrypellatectonae*	MFLUCC 12-0172b^T^	Shang et al. 2017	KY283085	KY421043
*Diatrypellaverruciformis*	UCROK1467	Lynch et al. 2013	JX144793	JX174093
*Diatrypellaverruciformis*	UCROK754	Lynch et al. 2013	JX144783	JX174083
*Diatrypellavulgaris*	HVFRA02	Trouillas et al. 2011	HQ692591	HQ692503
*Diatrypellavulgaris*	HVGRF03	Trouillas et al. 2011	HQ692590	HQ692502
*Diatrypellayunnanensis*	VT01	Zhu et al. 2021	MN653008	MN887112
*Eutypaarmeniacae*	ATCC 28120	Rolshausen et al. 2006	DQ006948	DQ006975
*Eutypaastroidea*	CBS 292.87	Rolshausen et al. 2006	AJ302458	DQ006966
*Eutypacamelliae*	HKAS 107022^T^	Samarakoon et al. 2022	MW240634	MW775593
*Eutypacerasi*	GMB0048^T^	Long et al. 2021	MW797104	MW814893
*Eutypacerasi*	GMB0049	Long et al. 2021	MW797105	MW814877
*Eutypalaevata*	E40C CBS 291.87	Acero et al. 2004	AJ302449	NA
*Eutypalata*	CBS290.87	Trouillas and Gubler, 2010	HM164736	HM164770
*Eutypalata*	EP18	Trouillas et al. 2011	HQ692611	HQ692501
*Eutypalata*	RGA01	Trouillas et al. 2011	HQ692614	HQ692497
*Eutypalejoplaca*	CBS 248.87	Rolshausen et al. 2006	DQ006922	DQ006974
*Eutypaleptoplaca*	CBS 287.87	Rolshausen et al. 2006	DQ006924	DQ006961
*Eutypamaura*	CBS 219.87	Rolshausen et al. 2006	DQ006926	DQ006967
*Eutypamicroasca*	BAFC 51550	Grassi et al. 2014	KF964566	KF964572
*Eutypasparsa*	3802 3b	Trouillas and Gubler, 2004	AY684220	AY684201
*Eutypatetragona*	CBS 284.87	Rolshausen et al. 2006	DQ006923	DQ006960
*Eutypellacaricae*	EL51C	Acero et al. 2004	AJ302460	NA
*Eutypellacearensis*	HUEFS 131070	de Almeida et al. 2016	KM396639	NA
*Eutypellacerviculata*	M68	Arhipova et al. 2012	JF340269	NA
*Eutypellacerviculata*	EL59C	Acero et al. 2004	AJ302468	NA
*Eutypellaleprosa*	EL54C	Acero et al. 2004	AJ302463	NA
*Eutypellaleprosa*	60	de Almeida et al. 2016	KU320622	NA
*Eutypellamicrotheca*	BCMX01	Paolinelli-Alfonso et al. 2015	KC405563	KC405560
*Eutypellamotuoensis*	FCATAS4035	Ma et al. 2023	OP538695	NA
*Eutypellamotuoensis*	FCATAS4082^T^	Ma et al. 2023	OP538693	OP557599
*Eutypellaparasitica*	CBS 210.39^T^	Jurc et al. 2006	DQ118966	NA
*Eutypellaquercina*	IRANC2543C^T^	Mehrabi et al. 2019	KX828139	KY352449
*Eutypellasemicircularis*	MP4669	Mehrabi et al. 2016	JQ517314	NA
*Eutypellatamaricis*	MFLUCC 14-0445	Thambugala et al. 2017	KU900330	KX453302
*Halocryptovalsasalicorniae*	MFLUCC 15-0185	Dayarathne et al. 2020b	MH304410	MH370274
*Halodiatrypeavicenniae*	MFLUCC 15-0953	Dayarathne et al. 2016	KX573916	KX573931
*Halodiatrypesalinicola*	MFLUCC 15-1277	Dayarathne et al. 2016	KX573915	KX573932
*Kretzschmariadeusta*	CBS 826.72	U’ren et al. 2016	KU683767	KU684190
*Monosporascuscannonballus*	CMM3646	Unpublished	JX971617	NA
*Monosporascuscannonballus*	ATCC:2693^T^	Unpublished	FJ430598	NA
*Neoeutypellabaoshanensis*	HMAS:255436	Phookamsak et al. 2019	NR164038	MH822888
*Paraeutypellacitricola*	HVVIT07	Trouillas et al. 2011	HQ692579	HQ692512
*Paraeutypellacitricola*	HVGRF01	Trouillas et al. 2011	HQ692589	HQ692521
*Paraeutypellaguizhouensis*	KUMCC 20-0017	Dissanayake et al. 2021	MW036141	MW239661
*Paraeutypellapseudoguizhouensis*	GMB0420 ^T^	Li et al. 2023	OP935186	OP938748
*Paraeutypellapseudoguizhouensis*	GMB0421	Li et al. 2023	OP935187	OP938749
*Paraeutypellavitis*	UCD2291AR	Úrbez-Torres et al. 2012	HQ288224	HQ288303
*Paraeutypellavitis*	UCD2428TX	Úrbez-Torres et al. 2009	FJ790851	GU294726
*Pedumisporarhizophorae*	BCC44877	Klaysuban et al. 2014	KJ888853	NA
*Pedumisporarhizophorae*	BCC44878	Klaysuban et al. 2014	KJ888854	NA
*Peroneutypaalsophila*	EL58C	Acero et al. 2004	AJ302467	NA
*Peroneutypacurvispora*	HUEFS 136877	de Almeida et al. 2016	KM396641	NA
*Peroneutypadiminutiasca*	MFLUCC 17-2144^T^	Shang et al. 2018	MG873479	MH316765
*Peroneutypadiminutispora*	HUEFS 192196	de Almeida et al. 2016	KM396647	NA
*Peroneutypahainanensis*	GMB0424^T^	Li et al. 2023	OP935179	OP938746
*Peroneutypahainanensis*	GMB0425	Li et al. 2023	OP935180	OP938747
*Peroneutypaindica*	NFCCI 4393^T^	Dayarathne et al. 2020a	MN061368	MN431498
*Peroneutypakochiana*	EL53M	Carmarán et al. 2006	AJ302462	NA
*Peroneutypakunmingensis*	HKAS 113189^T^	Phukhamsakda et al. 2022	MZ475070	MZ490589
*Peroneutypaleucaenae*	MFLU 18-0816^T^	Samarakoon et al. 2022	MW240631	MW775591
*Peroneutypalongiasca*	MFLU 17-1217^T^	Senwanna et al. 2017	MF959502	MG334558
*Peroneutypamackenziei*	MFLUCC 16-0072^T^	Shang et al. 2017	KY283083	KY706363
*Peroneutypamangrovei*	PUFD526	Phookamsak et al. 2019	MG844286	MH094409
*Peroneutypapolysporae*	NFCCI 4392^T^	Dayarathne et al. 2020a	MN061367	MN431497
*Peroneutypaqianensis*	GMB0431^T^	Li et al. 2023	OP935177	NA
*Peroneutypaqianensis*	GMB0432	Li et al. 2023	OP935178	NA
*Peroneutyparubiformis*	MFLUCC 17-2142^T^	Shang et al. 2018	MG873477	MH316763
*Pseudodiatrypehainanensis*	GMB0054^T^	Long et al. 2021	MW797111	MW814883
*Pseudodiatrypehainanensis*	GMB0055	Long et al. 2021	MW797112	MW814884
*Quaternariaquaternata*	EL60C	Acero et al. 2004	AJ302469	NA
*Quaternariaquaternata*	GNF13	Mehrabi et al. 2016	KR605645	NA
***Stromatolineagrisea***	**GMB4512**	**This study**	**PQ113920**	**PQ115208**
***Stromatolineagrisea***	**GMB450**8	**This study**	**PQ113921**	**PQ115209**
***Stromatolineaguizhouensis***	**GMB4523**	**This study**	**PQ113922**	**PQ115210**
***Stromatolineaguizhouensis***	**GMB451**5	**This study**	**PQ113923**	**PQ115211**
***Stromatolineahydei***	**GMB450**9	**This study**	**PQ113924**	**PQ115212**
***Stromatolineahydei***	**GMB453**8	**This study**	**PQ113925**	**PQ115213**
***Stromatolineahydei***	**GMB452**1	**This study**	**PQ113926**	**PQ115214**
*Stromatolinealinearis*	MFLUCC 11-0503	Dai et al. 2017	KU940150	-
*Stromatolinealinearis*	MFLUCC 15-0198	Dai et al. 2017	KU940149	MW775587
***Stromatolineaxishuiensis***	**GMB4535**	**This study**	**PQ113927**	**PQ115215**
***Stromatolineaxishuiensis***	**GMB4522**	**This study**	**PQ113928**	**PQ115216**
***Stromatolineaxishuiensis***	**GMB451**4	**This study**	**PQ113929**	**PQ115217**
*Vasilyeva cinnamomi*	GMB0418^T^	Li et al. 2023	OP935174	OP938737
*Vasilyeva cinnamomi*	GMB0419	Li et al. 2023	OP935175	OP938738
*Xylariahypoxylon*	CBS 122620	Peršoh et al. 2009	AM993141	KX271279

The Bayesian inference analysis was performed in MrBayes v. 3.2.1 (Ronquist et al. 2012). The model of evolution was estimated by MrModeltest 2.2 (Nylander 2004). The Markov chain Monte Carlo (MCMC) sampling in MrBayes v.3.2.2 (Ronquist et al. 2012) was used to determine the posterior probabilities (PP). Six simultaneous Markov chains were run for 1,000,000 generations, and trees were sampled every 1000^th^ generation. The phylogenetic tree was visualized in FIGTREE v.1.4.3 (Rambaut 2012). All analyses were run on the CIPRES Science Gateway v 3.3 web portal (Miller et al. 2010).

## ﻿Results

### ﻿Phylogenetic analyses

**Figure 1.**
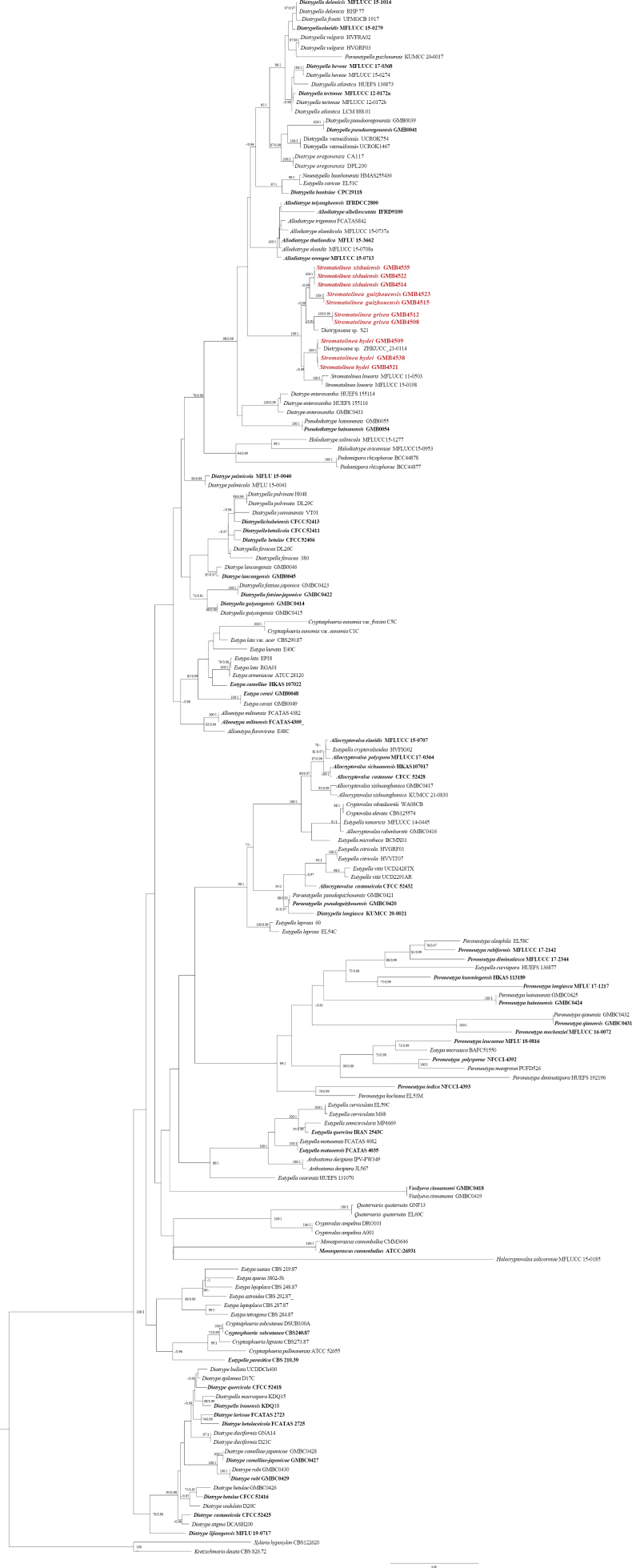
**A–C.** Phylogenetic tree generated from maximum likelihood analysis (RAxML) based on combined ITS and TUB2 sequences data. Bootstrap support values for maximum likelihood (ML) greater than 70% and Bayesian posterior probabilities (BPP) greater than 0.90 are displayed above or below the respective branches (ML/BPP). The species obtained in this study are in red and ex-type taxa are in bold.

After the exclusion of ambiguously aligned regions and long gaps, the final combined data matrix contained 1,450 characters. *Kretzschmariadeusta* (CBS 826.72) and *Xylariahypoxylon* (CBS 122620) were added as the outgroup. The tree topology derived from Maximum Likelihood (ML) analysis closely resembled that of Bayesian Inference (BI) analysis. The best-scoring RAxML tree is shown in Fig. 1. The topology of the phylogenetic tree is similar to those in previous studies (Li et al. 2023; Ma et al. 2023). The new genus *Stromatolinea*, including four species, formed a distinct clade that represents its monophyletic status. The strains ZHKUCC 21-0114 and S21 clustered in the clade of the new genus, are deposited in NCBI under the name *Diatrypella* sp., but remain unpublished. The details provided in NCBI for these two strains (ZHKUCC 21-0114 and S21) were searched, but no records were found.

### ﻿Taxonomy

#### ﻿ Stromatolinea

Taxon classificationFungiXylarialesDiatrypaceae

K. Habib & Q.R. Li gen. nov.

2527684A-300D-5B81-BA95-1C7363C8CFF9

853267

##### Etymology.

Referring to linear characteristics of the stromata.

##### Type species.

*Stromatolineahydei* K. Habib & Q.R. Li, sp. nov.

##### Description.

Saprobic on dead bamboo culms, forming black parallel elongate ascostromata on the host, surrounded by grey or black patches like pseudostromata. **Pseudostromata** grey or black, spreading between stromata and across the host surface. **Sexual morph: Stromata** parallel elongate, linear, consistent to inconsistent in thickness, fusiform, high, solitary to confluent, slit to non-slit, black or grey on its sides, exposing black ostioles. Upper cells of stromata near the perithecial ostiole black, thick-walled. **Stromatic tissue** completely yellow or yellow above and white between/below perithecia, compact. **Ascomata** perithecial, few to frequent, immersed in stromata, globose to subglobose, ostiolate centrally, with a neck, opening to outer surface, slight erumpent over stromata, appearing as black shinny spots. **Peridium** composed of elongate cell, ***texture angularis***, outer thick layer, dark brown, inner hyaline, surrounded by yellow or white and yellow stromatic tissue. **Hamathecium** paraphyses, filiform, hyaline, long. **Asci** 8-spored, clavate, with a long and thin pedicel, apically rounded to truncate, J- apical ring. **Ascospores** allantoid, aseptate, straight to slightly curved, rounded at both ends, subhyaline, with oil droplets in both ends. **Asexual morph**: undetermined.

##### Notes.

Phylogenetically, *Eutypa* is polyphyletic (Fig. 1), a species distributed in different clades. Ma et al. (2023) proposed a new genus, *Alloeutypa*, which exhibits close affinity to *Eutypa*. However, based on the presence of *Diatrype*-like discrete stromata with yellowish-green interior tissue characteristics and forming separate monophyletic clades, they proposed *Alloeutypa* as a new genus to accommodate *Alloeutypamilinensis* and *A.flavovirens* (Ma et al. 2023).

Morphologically, *Stromatolinea* is similar to *Alloeutypa*, as both possess yellowish-green interior tissue. However, the new genus is distinguished from *Alloeutypa* by its linear stromata and phylogenetically, they are clustered very distantly. The strains of *Stromatolinea* form a monophyletic clade representing its distinct position. Thus, based on morphological evidence and phylogenetic analyses, we accommodate *Stromatolinea* as a new genus with *Stromatolineahydei* designated as the type species.

#### ﻿ Stromatolinea grisea

Taxon classificationFungiXylarialesDiatrypaceae

K. Habib & Q.R. Li sp. nov.

3342D4C1-3A1A-5487-8F65-3F3604786BFB

853270

Fig. 2

##### Type.

• China, Guizhou Province, Xishui Country, Changjian Gully, (28°19'58″N, 106°11'50″E), altitude: 1,180 m, subtropical forest, on dead culms of *Phyllostachys* sp., 27 December 2023, Xin Zhou, R-17, (Holotype, GMB4512; ex-type, GMBC4512; isotype, KUN-HKAS133213).

**Figure 2.**
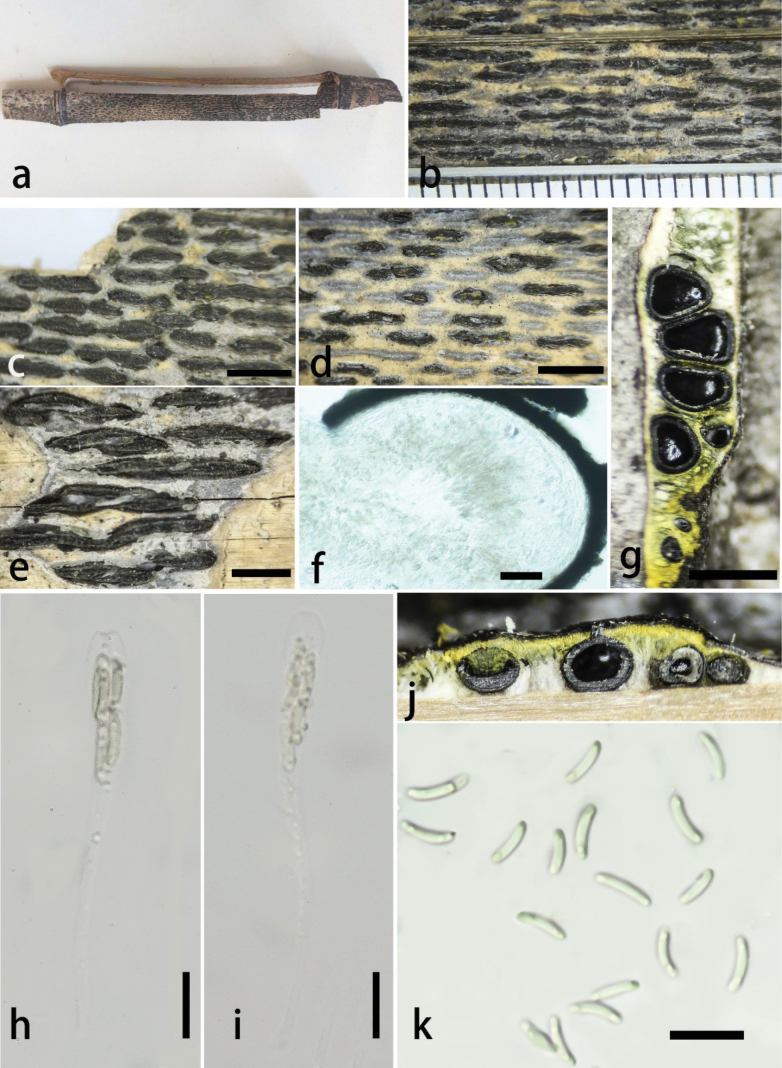
*Stromatolineagrisea* (GMB4512) **a** habitat of a type material **b–e** appearance of stromata and pseudostromata on bamboo host **f** peridium of ascoma **g** horizontal section of ascostromata **h, i** asci **j** vertical sections of ascomata in stroma **k** ascospores. Scale bars: 1 mm (**b**); 3 mm (**c–e**); 30 μm (**f**); 0.4 mm (**g, j**); 20 μm (**h, i**); 10 μm (**k**).

##### Etymology.

The epithet “*grisea*” refers to the grey color of pseudostromata.

##### Description.

Saprobic on dead culms of *Phyllostachys* sp., forming black parallel elongate ascostromata on the host, surrounded by grey patches like pseudostromata. **Pseudostromata** grey, spreading between stromata and across the host surface. **Sexual morph**: **Stromata** 2–15 mm long, 400–800 μm wide, 400–600 μm high, parallel elongate, inconsistent in thickness, thin in between, fusiform, solitary to confluent, non-slit, black, exposing black ostioles. Upper cells of stromata near the perithecial ostiole black, thick-walled. Stromatic tissue yellow above and white between/below perithecia, compact. **Ascomata** 250–420 μm wide, 260–450 μm high, perithecial, 2–5 per stromata, usually 2 or 3 per stromata, immersed in stromata, globose to subglobose, ostiolate centrally, with a neck, opening to outer surface, 80–100 × 35–60 μm, slight erumpent over stromata, appearing as black shinny spots. **Peridium** 15–30 μm thick, cell elongate, ***texture angularis***, outer thick layer dark brown, inner hyaline, surrounded by yellow stromatic tissue. **Hamathecium** paraphyses, filiform hyaline, 50–73 × 1–3.2 μm. **Asci** 50–95 × 5.5–9.8 μm (x̄ = 73 × 6.2 μm, n = 30), 8-spored, unitunicate, clavate, with a long and thin pedicel, apically rounded to truncate, J- apical ring. **Ascospores** 5.8–8.2 × 1.4–2 μm (x̄ = 7.5 × 1.6 μm, n = 30), allantoid, aseptate, straight to slightly curved, rounded at both ends, subhyaline, single oil droplets in both ends. **Asexual morph**: Undetermined.

##### Culture characteristics.

Ascospores germinating on PDA within 24 hours, colonies on PDA, white when young, became pale, thinning toward the edge, white from above, reverse pale, no pigmentation, and no sporulation produced on the PDA medium.

##### Addition material examined.

• China, Guizhou Province, Xishui Country, Changjian Gully, (28°19'56″N, 106°11'48″E), altitude: 1,180 m, subtropical forest, on dead culms of *Phyllostachys* sp., 2 January 2024, Lili Liu & Yan Lin, R-200 (GMB4508).

##### Notes.

It is morphologically similar to *Stromatolineahydei* and *S.xishuiensis* but can be easily distinguished by the appearance of stromata and pseudostromata color. The pseudostromata of the latter two species are black, whereas grey in *S.grisea*. The stromata of *S.hydei* and *S.xishuiensis* are consistent in thickness and possess frequent ascomata, whereas the stromata of *S.grisea* are inconsistent in thickness, thin in between, and possess usually 2 or 3 ascomata. Moreover, stromatic tissue is yellow above and white between or below perithecia in *S.grisea*, while it is completely yellow in *S.hydei* and *S.xishuiensis*. The comparison of ITS sequences revealed 94% and 98% similarity to *S.hydei* and *S.xishuiensis*, respectively, while TUB2 sequences displayed 93% and 94% similarity to *S.hydei* and *S.xishuiensis*, respectively. Differentiation from other known species of the genus is discussed in the note section of the below described species.

#### ﻿ Stromatolinea guizhouensis

Taxon classificationFungiXylarialesDiatrypaceae

K. Habib & Q.R. Li sp. nov.

87498C4A-F59A-5D0B-85AF-BEFC11BFD26B

853272

Fig. 3

##### Type.

• China, Guizhou Province, Anshun City, Pingba County (26°15'11″N, 105°56'51″E), altitude: 1,102 m, subtropical forest, on dead culms of *Phyllostachys* sp., 25 August 2023, JWS-28 (Holotype, GMB4523; ex-type, GMBC4523; isotype, KUN-HKAS133214).

**Figure 3.**
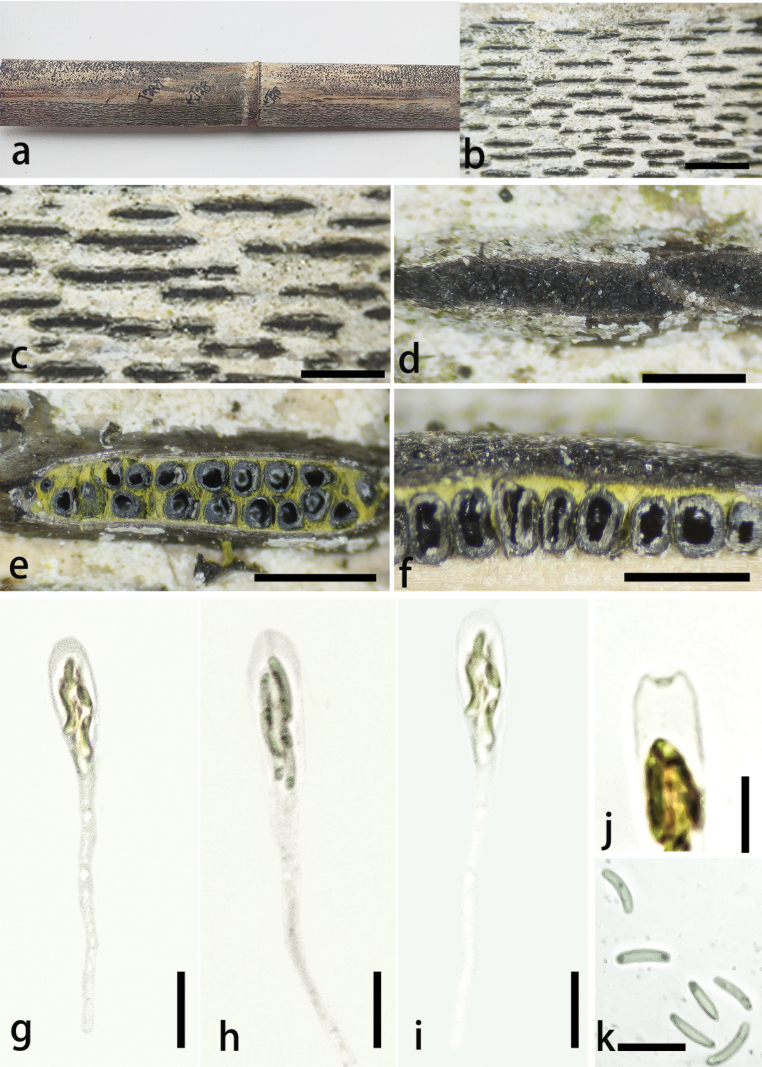
*Stromatolineaguizhouensis* (GMB4523) **a** habitat of material **b, c** appearance of stromata on host **d** erumpent ostiole **e** horizontal section of ascostromata **f** vertical sections of ascomata in stroma **g-i** asci **j** apical ring of ascus **k** ascospores. Scale bars: 3 mm (**b, c**); 1 mm (**d–f**); 20 μm (**g–j**); 10 μm (**k**).

##### Etymology.

The epithet “*guizhouensis*” refers to the locality of the collection, Guizhou province.

##### Description.

Saprobic on dead culms of *Phyllostachys* sp., forming black parallel elongate ascostromata on the host. **Pseudostromata** absent. **Sexual morph**: **Stromata** 2–8.5 mm long, 350–800 μm wide, 400–600 μm high, parallel elongate, consistent in thickness, linear, long fusiform, solitary, sometime confluent, non-slit, distinctly gray at sides, often overlain by white crystalline, black at center, exposing black ostioles. Upper cells of stromata near the perithecial ostiole black, thick-walled. Stromatic tissue yellow between and beneath perithecia, compact. **Ascomata** 150–250 μm wide, 250–420 μm high, perithecial, frequent, 10–25 per stromata, immersed, linearly arranged, obpyriform, ostiolate centrally, with a neck, opening to outer surface, 70–90 × 35–60 μm, slight erumpent over stromata, appearing as black spots, slight shinny. **Peridium** 5–10 μm thick, cell elongate, ***texture angularis***, outer thick layer dark brown, inner hyaline, surrounded by yellow stromatic tissue. **Hamathecium** paraphyses, filiform, hyaline, 54–70 × 1–3.2 μm. **Asci** 55–100 × 5.5–8 μm (x̄ = 67.2 × 6.8 μm, n = 30), 8-spored, unitunicate, clavate, with a long and thin pedicel, apically rounded to truncate, J- apical ring. **Ascospores** 5.8–9 × 1–2 μm (x̄ = 7.6 × 1.5 μm, n = 30), allantoid, aseptate, straight to slightly curved, rounded at both ends, subhyaline, smooth-walled, single oil droplets in both ends. **Asexual morph**: Undetermined.

##### Culture characteristics.

Ascospores germinating on PDA within 24 hours, colonies on PDA, white when young, became pale, dense at centre, thinning toward the edge, reverse white at the margin, pale at the centre, no pigmentation, and no sporulation produced on the PDA medium.

##### Additional material examined.

• China, Guizhou Province, Huaxi District, Xiaohe Village, China (26°29'29″N, 106°42'09″E), altitude: 1,097 m, subtropical forest, on dead culms of *Phyllostachys* sp., 2 January 2024, Xin Zhou & W.Y. Zeng, H-8 (GMB4515).

##### Notes.

Morphologically, *Stromatolineaguizhouensis* is similar to *Stromatolinealinearis* (= *Diatrypephaselinoides* Rappaz; *Eutypalinearis* Rehm), both exhibiting parallel elongate fusiform stromata with yellow stromatic tissue. However, it differs from *S.linearis* in having non-slit stromata, distinctly grey at sides, overlain by white crystalline material (Fig. 3b, c) and slightly larger ascospores (5.8–9 μm, x̄ = 7.6 μm), compared to *S.linearis* with longitudinally slit stromata when mature and smaller ascospores (5–7 μm, x̄ = 6.1 μm) (Rehm 1907; Dai et al. 2017). From the other newly described species, it lacks pseudostromata and exhibits grey stromata overlain by white crystalline material.

Another morphologically similar species, *Alloeutypamilinensis* also features yellow stromatic tissue but can be easily differentiated by its stromata morphology. *Alloeutypamilinensis* exhibits scattered oblong to strip-shaped stromata measuring 0.9–2.2 mm in width and with larger ascospores (6.6–10.1 × 1.7–2.6 μm, x̄ = 8.5 × 2.1 μm) (Ma et al. 2023).

#### ﻿ Stromatolinea hydei

Taxon classificationFungiXylarialesDiatrypaceae

K. Habib & Q.R. Li sp. nov.

90C412CB-8C81-5FDC-899B-3E058D81E058

853274

Fig. 4

##### Type.

• China, Guizhou Province, Anlong County Suburban (25°05'56″N, 105°26'34″E), altitude: 856 m, subtropical forest, on dead culms of *Phyllostachys* sp., 23 September 2023, Youpeng Wu, JWS-8 (Holotype, GMB4509; ex-type, GMBC4509; isotype, KUN-HKAS133215).

**Figure 4.**
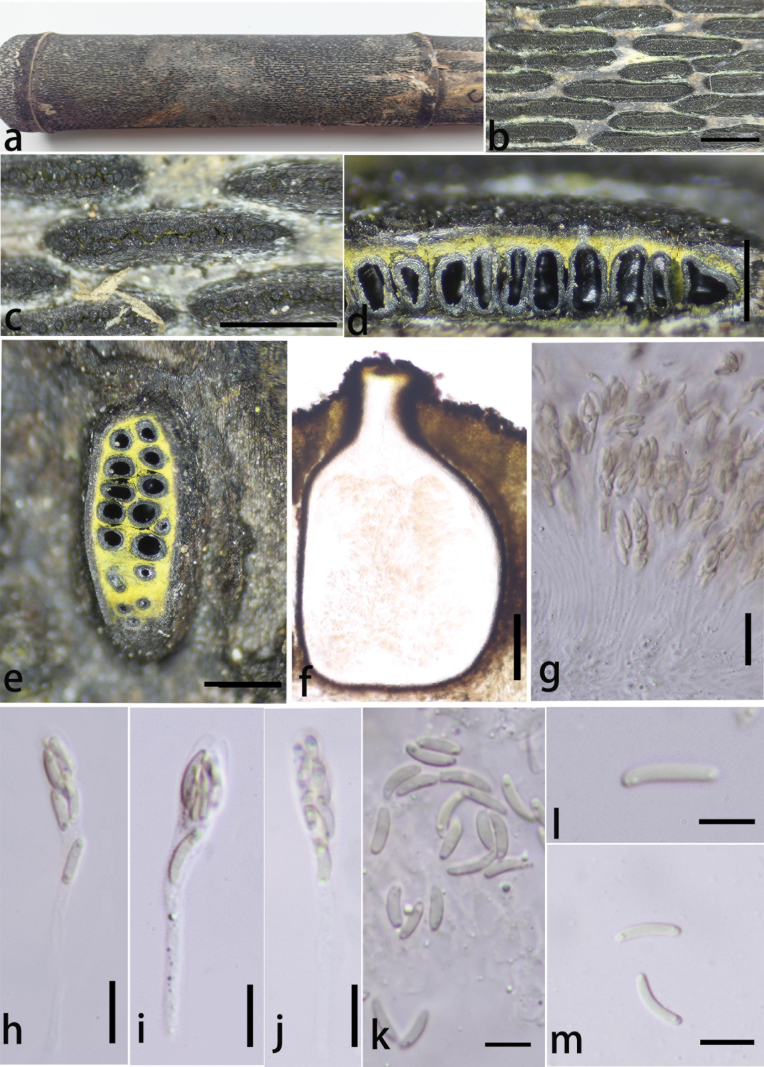
*Stromatolineahydei* (GMB4509) **a** habitat of material **b, c** appearance of stromata on host **d** vertical section of ascomata in stroma **e** horizontal section of ascostromata **f** vertical section of ascoma **g–j** asci **k–m** ascospores. Scale bars: 3 mm (**b, c**); 0.5 mm (**d, e**); 100 μm (**f**); 15 μm (**g–j**); 5 μm (**k–m**).

##### Etymology.

The epithet “*hydei*” pays tribute to the renowned mycologist, Prof. Kevin David Hyde, in recognition of his valuable contributions to the field of mycology.

##### Description.

Saprobic on dead culms of *Phyllostachys* sp., forming black parallel elongate ascostromata on the host, surrounded by black patches like pseudostromata. **Pseudostromata** black, spreading between stromata and across the host surface forming the darkened region. **Sexual morph**: **Stromata** 2–10 mm long, 400–800 μm wide, 400–620 μm high, parallel elongate, straight, long fusiform, solitary, sometime confluent, slit when mature, black, above plane, exposing black ostioles. Upper cells of stromata near the perithecial ostiole black, thick-walled. Stromatic tissue yellow between and beneath perithecia, compact. **Ascomata** 150–270 μm wide, 260–440 μm high, perithecial, frequent, 10–25 per stromata, immersed in stromata, linearly arranged, obpyriform, ostiolate centrally, with a neck, opening to outer surface, 80–100 × 35–60 μm, slight erumpent over stromata, appearing as black spots, slight shinny. **Peridium** 5–10 μm thick, cell elongate, ***texture angularis***, outer thick layer dark brown, inner hyaline, surrounded by yellow stromatic tissue. **Hamathecium** paraphyses, filiform. hyaline, 50–68 × 1–3.5 μm. **Asci** 50–80 × 5.5–8 μm (x̄ = 64 × 6.5 μm, n = 15), 8-spored, unitunicate, clavate, with a long and thin pedicel, apically rounded to truncate, a J- apical ring. **Ascospores** 5.8–10 × 1.4–2.5 μm (x̄ = 8 × 1.8 μm, n = 20), allantoid, aseptate, straight to slightly curved, rounded at both ends, subhyaline, single oil droplets in both ends. **Asexual morph**: Undetermined.

##### Culture characteristics.

Ascospores germinating on PDA within 24 hours, colonies on PDA, white when young, became pale, dense at centre, thinning toward the edge, reverse pale-white, no pigmentation, and no sporulation produced on the PDA medium.

##### Addition material examined.

China • Guizhou Province, Xishui Country, Changjian Gully, (28°19'57″N, 106°11'32″E), altitude: 1,180 m, subtropical forest, on dead culms of *Phyllostachys* sp., 2 January 2024, Lili Liu & Yan Lin, R-27 (GMB4538) • China, Guizhou Province, Xishui Country, Changjian Gully, (28°19'51″N, 106°11'49″E), altitude: 1,185 m, subtropical forest, on dead culms of *Phyllostachys* sp., 2 January 2024, Xin Zhou, R-4 (GMB4521).

##### Notes.

*Stromatolineahydei* is morphologically similar to *S.linearis* (=*Diatrypephaselinoides*; *Eutypalinearis*), both displaying parallel elongate fusiform stromata with yellow stromatic tissue and slit mature stromata. However, *S.hydei* is distinguishable by its stromata, which are surrounded by black patches resembling pseudostromata, which spread between stromata and across the host surface, forming a darkened region. Additionally, *S.hydei* has larger ascospores 5.8–10 × 1.4–2.5 μm (with an average of 8 × 1.8 μm), compared to *S.linearis*, whose ascospores range from 5–7 × 1–2 μm, with an average of 6.1 × 1.4 μm (Dai et al. 2017).

#### ﻿ Stromatolinea xishuiensis

Taxon classificationFungiXylarialesDiatrypaceae

K. Habib & Q.R. Li sp. nov.

41B1CEE4-3D0E-5B33-9979-2B419C7F9D58

853275

Fig. 5

##### Type.

• China, Guizhou Province, Xishui Country, Changjian Gully, (28°19'58″N, 106°11'50″E), altitude: 1,180 m, subtropical forest, on dead culms of *Phyllostachys* sp., 27 December 2023, Xin Zhou, R-7 (Holotype, GMB4535; ex-type, GMBC4535; isotype, KUN-HKAS133216).

**Figure 5.**
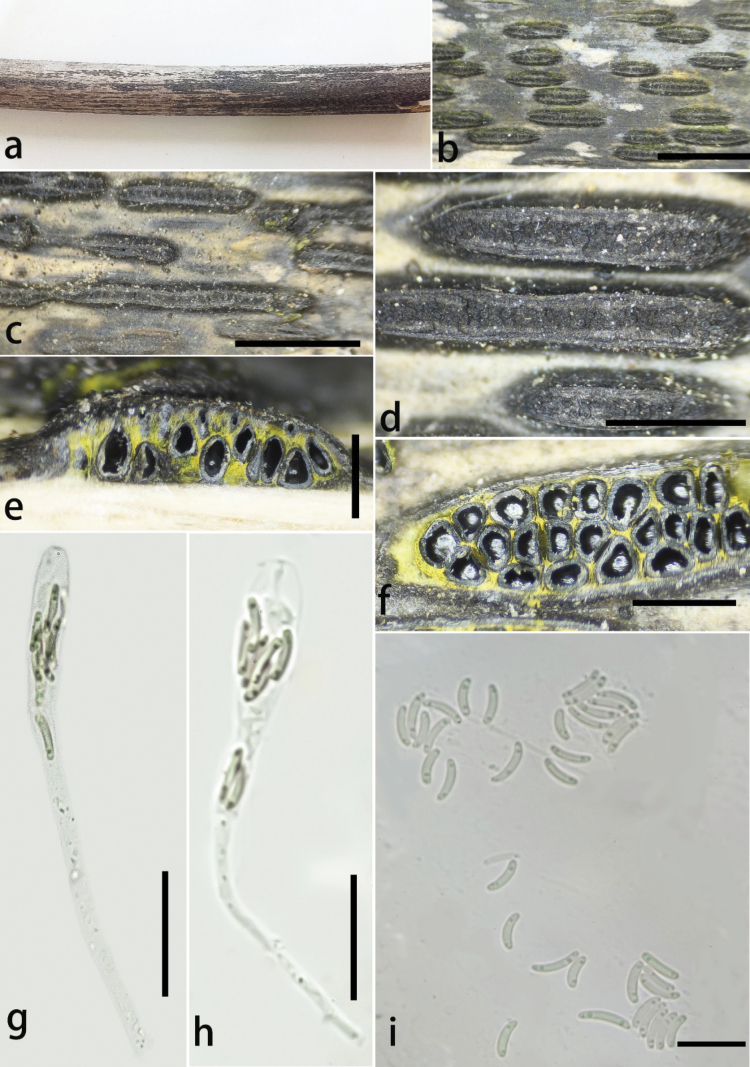
*Stromatolineaxishuiensis* (GMB4535) **a** habitat of material **b–d** appearance of stromata on host **e** vertical section of ascomata in stroma **f** horizontal section of ascostromata **g, h** asci **i** ascospores. Scale bars: 3 mm (**b, c**); 1 mm (**d**); 0.5 mm (**e, f**); 20 μm (**g, h**); 10 μm (**i**).

##### Etymology.

The epithet “*xishuiensis*” refers to the locality of the collection, Xishui County.

##### Description.

Saprobic on dead bamboo culms, forming black parallel elongate ascostromata on the host, surrounded by black patches like pseudostromata. **Pseudostromata** black, spreading between stromata and across the host surface forming the darkened region. **Sexual morph**: **Stromata** 2–10 mm long, 350–600 μm wide, 400–550 μm high, parallel elongate, consistent in thickness, straight, long fusiform, solitary to confluent, non-slit, black, exposing black ostioles. Upper cells of stromata near the perithecial ostiole black, thick-walled. Stromatic tissue yellow between and beneath perithecia, compact. **Ascomata** perithecial, 150–220 μm wide, 240–300 μm high, frequent, 10–28 per stromata, immersed in stromata, obpyriform, irregular arranged, ostiolate centrally, with a neck, opening to outer surface, slight erumpent over stromata, appearing as black spots. **Peridium** 5–15 μm thick, cell elongate, texture angularis, outer thick layer dark brown, inner hyaline, surrounded by yellow stromatic tissue. **Hamathecium** paraphyses, hyaline, 50–70 × 1–3.6 μm, filiform. **Asci** 60–90 × 5.5–8 μm (x̄ = 70 × 7 μm, n = 15), 8-spored, clavate, with a long and thin pedicel, apically rounded to truncate, J- apical ring. **Ascospores** 5.8–8.2 × 1–2.2 μm (x̄ = 7.1 × 1.4 μm, n = 20), allantoid, aseptate, straight to slightly curved, rounded at both ends, subhyaline, 1–2 oil droplets in both ends. **Asexual morph**: Undetermined.

##### Culture characteristics.

Ascospores germinating on PDA within 24 hours, colonies on PDA, white when young, pale and dense at centre, thinning toward the edge, reverse pale-white, no pigmentation, and no sporulation produced on the PDA medium.

##### Additional material examined.

• China, Guizhou Province, Zunyi City, Suiyang Country, Kuankuoshui National Nature Reserve (28°29'33.64"N, 107°9'23.66"E), altitude: 1,634 m, subtropical forest, on dead culms of *Phyllostachys* sp., 23 September 2023, Qirui Li, K3N (GMB4522) • China, Guizhou Province, Zunyi City, Xishui Country, Changjian Gully, (28°19'58″N, 106°11'54″E), altitude: 1,180 m, on dead culms of *Phyllostachys* sp., 27 December 2023, Xin Zhou, R-5, (GMB4514).

##### Notes.

*Stromatolineaxishuiensis* can be distinguished from *S.guizhouensis* and *S.linearis* by its stromata surrounded by black patches spread between the stromata and across the host surface, forming a darkened region. Moreover, its ascomata are irregularly arranged in stroma. Morphologically, it is most similar to *S.hydei*, which also exhibits black patches spreading between stromata and across the host surface. However, *S.hydei* has slightly wider stromata, measuring 400–800 μm wide and 400–620 μm high, linearly arranged larger ascomata, measuring 150–270 μm wide and 260–440 μm high, and bigger ascospores, measuring 5.8–10 × 1.4–2.5 μm (average 8 × 1.8 μm). The ITS and β-tubulin sequence data of *S.xishuiensis* and *S.hydei* demonstrates 94% and 95% similarity, respectively.

#### ﻿ Stromatolinea linearis

Taxon classificationFungiXylarialesDiatrypaceae

(Rehm) K. Habib & Q. R. Li comb. nov.

6B1F2DCC-9078-53F7-B4AE-BEBDFE8B7C14

853282

Eutypa linearis Rehm, Annls mycol. 5(6): 523 (1907) (Basionym). Synonym
= Diatrypephaselinoides Rappaz, Mycol. helv. 2(3): 442 (1987). Synonym

##### Description.

See Dai et al. (2017).

##### Notes.

The fungus was originally documented by Rehm (1907) from a specimen collected in Brazil, *Eutypalinearis* underwent a taxonomic revision by Rappaz (1987), who reclassified it as *Diatrypephaselinoides* (non *Diatrypelinearis* Ellis & Everh. 1897). Dai et al. (2017) provided molecular data and a reference specimen of this taxon. It is characterized by well-developed linear stromata with yellow-green interior tissue, long-stipitate asci, with hyaline to subhyaline allantoid ascospores, measuring 5–7 × 1.5–1.8 μm (Rappaz 1987; Dai et al. 2017). Phylogenetically, it clusters together with other *Stromatolinea* species in a distinct clade. The morphological character of this taxon also aligns with those of *Stromatolinea*, providing compelling support for its placement within the *Stromatolinea* taxonomic framework.

#### ﻿ Stromatolinea phaselina

Taxon classificationFungiXylarialesDiatrypaceae

(Mont.) K. Habib & Q. R. Li comb. nov.

34C2DE0F-2F3F-58AF-8F75-E42E1841DBD9

855036

Sphaeria phaselina Mont., Ann. Sci. Nat., Bot., sér. 4 3: 129 (1855) (Basionym). Synonym.
= Diatrypephaselina (Mont.) Rappaz, Mycol. Helv. 2(3): 442 (1987). Synonym.^1^

##### Notes.

*Stromatolineaphaselina* was first described and illustrated by Montagne in 1855 based on a collection from Guyana. Rappaz (1987) conducted a detailed analysis of various species that had been reported under different names (given above as synonyms). He reviewed their descriptions and type materials, though some of type material were lost. Rappaz (1987) synonymized *E.kusanoi* and *E.bambusina* with *Eutypahypoxantha* and grouped these along with *Sphaeriaphaselina* and *Eutypellahypoxantha* under the broader name *Diatrypephaselina* (Mont.) Rappaz. The morphological character of this taxon also aligns with those of *Stromatolinea*, providing compelling support for its placement within the *Stromatolinea* taxonomic framework.

Morphologically, *Stromatolineaphaselina* resembles *S.grisea* in having yellow entostromatic tissue above and white tissue between or below the perithecia. However, there are no reports of pseudostromata presence, detailed stromata morphology, or the number of perithecia per stromata in the published description of *Diatrypephaselina* (Rappaz 1987). Furthermore, descriptions and synonymized accounts of this species report very short asci sizes, measuring 25–35 × 5–7 μm. This is problematic because the family is known to typically possess long asci. This gap in detailed morphological data limits our ability to fully understand and differentiate *Diatrypephaselina* from other species. Without a detailed description and access to type material or DNA data, we cannot definitively classify it within the key of the genus.

The synonyms of this species are not updated in Index Fungorum and Mycobank, where they are still listed as separate species. Given Rappaz (1987) thorough analysis of historical descriptions and most of the original materials, we consider his classification/synonyms of the species to be well-founded and reliable.

## ﻿Discussion

The generic concepts of Diatrypaceae have been unstable; several new genera within the family have been reported through a combination of morphological characteristics and multi-locus phylogeny. Early classification systems of Diatrypaceae were mainly based on stromatal features including the degree of stromatal development, structure of perithecial necks, and type of host tissue (Glawe and Jacobs 1987; Rappaz 1987). However, the morphological variability of stromata has caused significant confusion within Diatrypaceae. Many genera, including *Neoeutypella*, *Allodiatrype*, *Diatrype*, *Diatrypella*, *Allocryptovalsa*, *Cryptovalsa*, *Eutypella*, and *Paraeutypella*, exhibit similar stromatal characteristics, limiting their utility for species identification (Li et al. 2023). This confusion has led to polyphyletic genera, where species have often been transferred between genera (Shang et al. 2017, 2018; Phookamsak et al. 2019; Konta et al. 2020; Ma et al. 2023). In this study, we introduce a new genus that phylogenetically forms a well-supported distinct clade and morphologically distinguished by its linear stromata and yellow interior tissue. This new genus includes four new species namely *S.grisea*, *S.guizhouensis*, *S.hydei*, and *S.xishuiensis*. Additionally, *Stromatolinealinearis* and *S.phaselina* are proposed for *Eutypalinearis* and *D.phaselina*, respectively, based on morphological characteristics and comparative analysis.

Species within *Stromatolinea* can be differentiated by key morphological features, including the presence or absence and color of pseudostromata; stromata size, color, slit presence, and interior tissue color; ascomata number, arrangement, and measurements; and ascospores dimensions. Furthermore, significant phylogenetic distances in the ITS and TUB2 regions also serve as valuable tools for species discrimination. Notably, all *Stromatolinea* species have been reported as saprobes on dead bamboo, implying a potential host specificity confined to bamboo.

### ﻿Key to species

**Table:** 

1	Pseudostromata absent	**2**
–	Pseudostromata well-developed	**3**
2	non-slit stromata, distinctly grey at sides, ascospores 5.8–9 μm long, averaging = 7.6 μm	***S.guizhouensis***
–	stromata slit when mature, color black, ascospores 5–7 μm long, averaging = 6.1 μm	***S.linearis***
3	grey pseudostromata, stromata inconsistent in thickness, possess few ascomata, interior tissue yellow above and white between or below perithecia, ascospores 5.8–8.2 × 1.4–2 μm, averaging = 7.5 × 1.6 μm	***S.grisea***
–	black pseudostromata, stromata consistent in thickness	**4**
4	stromata 400–800 μm wide, slit when mature, ascomata linearly arranged, 150–270 μm wide and 260–440 μm high, ascospores 5.8–10 × 1.4–2.5 μm, averaging = 8 × 1.8 μm	***S.hydei***
–	stromata 350–600 μm wide, non-slit, ascomata irregularly arranged, 150–220 μm wide and 240–300 μm high, ascospores 5.8–8.2 × 1–2.2 μm, averaging = 7.1 × 1.4 μm	***S.xishuiensis***

## Supplementary Material

XML Treatment for Stromatolinea

XML Treatment for Stromatolinea grisea

XML Treatment for Stromatolinea guizhouensis

XML Treatment for Stromatolinea hydei

XML Treatment for Stromatolinea xishuiensis

XML Treatment for Stromatolinea linearis

XML Treatment for Stromatolinea phaselina

## Footnotes

See Rappaz (1987) for other synonyms and description.

## ﻿Additional information

### Author ORCIDs

Kamran Habib https://orcid.org/0000-0003-2572-0306

Hongmin Hu https://orcid.org/0000-0003-3894-3269

Qianzhen Wu https://orcid.org/0000-0001-9992-8404

Jichuan Kang https://orcid.org/0000-0002-6294-5793

Qirui Li https://orcid.org/0000-0001-8735-2890

### Data availability

The datasets generated during and/or analyzed during the current study are available in the MycoBank repository (included in the manuscript), and GenBank (included in Table 1). And also, the datasets generated during and/or analyzed during the current study are available from the corresponding author on reasonable request.

## Supplementary materials

Supplementary material 1 Alignment file Data type fasta https://binary.pensoft.net/file/1128034This dataset is made available under the Open Database License (http://opendatacommons.org/licenses/odbl/1.0/). The Open Database License (ODbL) is a license agreement intended to allow users to freely share, modify, and use this Dataset while maintaining this same freedom for others, provided that the original source and author(s) are credited.Kamran Habib, Xin Zhou, Wenyu Zeng, Xu Zhang, Hongmin Hu, Qianzhen Wu, Lili Liu, Yan Lin, Xiangchun Shen, Jichuan Kang, Qirui Li
